# Fatigue Level Associated with Quality of Life for Prostate Cancer Patients: Results from the All of Us Research Program

**DOI:** 10.3390/cancers17091531

**Published:** 2025-04-30

**Authors:** Hui-Yi Lin, Masuma Mannan, Yu-Wen Chiu, Ya-Hsin Li, Rajasree P. Chowdry, Tung-Sung Tseng

**Affiliations:** 1 School of Public Health, Louisiana State University Health Sciences Center, New Orleans, LA 70112, USA; mmanna@lsuhsc.edu (M.M.); ychiu@lsuhsc.edu (Y.-W.C.); ttseng@lsuhsc.edu (T.-S.T.); 2 Department of Health Policy and Management, Chung-Shan Medical University, Taichung City 40201, Taiwan; yli@csmu.edu.tw; 3 School of Medicine, Louisiana State University Health Sciences Center, New Orleans, LA 70112, USA; rchow1@lsuhsc.edu

**Keywords:** quality of life, fatigue, prostate cancer, physical health, mental health

## Abstract

Background/Objectives: Prostate cancer (PCa) patients frequently experience extended survivorship, highlighting the importance of quality of life (QoL) in cancer care. QoL has been shown to influence survival and PCa progression. For PCa patients, fatigue is a common issue due to cancer itself or its treatments. However, many QoL studies on PCa patients are limited by small sample sizes in clinic settings and often overlook the impact of fatigue and health-related behaviors. To fill this gap, this study aims to evaluate fatigue associated with QoL and related health measures among PCa patients. Methods: This study used the All of Us cohort with 6426 PCa patients. Factors associated with QoL and health outcomes were tested using logistic models. Results: Among PCa patients, 8.8% reported poor QoL, 20.9% reported poor physical health, and 9.0% reported poor mental health. Around 24.5% and 5.0% of PCa patients had a moderate and high fatigue level, respectively. A high fatigue level was significantly associated with poor QoL (OR = 2.33, *p* < 0.001), poor physical health (OR = 8.20, *p* < 0.001), and poor mental health (OR = 3.62, *p* < 0.001), adjusting for other factors. Social function ability also played a critical role in QoL and health outcomes. In addition, PCa therapy was associated with a higher risk of poor physical health (OR = 1.30, *p* = 0.030) but not associated with QoL and mental health. Conclusions: This study provides valuable insights about fatigue’s important role in QoL, physical health, and mental health for PCa patients. Effective fatigue management is suggested to enhance QoL and overall well-being for PCa patients.

## Simple Summary

Prostate cancer (PCa) patients often live longer, so this makes their quality of life (QoL) important. PCa patients usually experience fatigue because of the cancer itself or its treatments. This study evaluated the relationship between fatigue, QoL, and health outcomes among 6426 PCa patients. It found that 8.8% reported poor QoL, 20.9% had poor physical health, and 9.0% reported poor mental health. Around 24.5% experienced moderate fatigue, and 5.0% reported high fatigue levels. Higher fatigue levels were associated with poorer QoL, poor physical health, and poor mental health, adjusting for other factors. Social function ability also played an important role in QoL and health outcomes. Additionally, PCa treatment increased the risk of poor physical health but did not affect QoL or mental health. The findings highlight the importance of managing fatigue for improving the overall well-being and clinical outcomes of PCa patients.

## 1. Introduction

PCa is the leading cause of cancer incidence and the second leading cause of cancer deaths among American men in 2024 [1]. Approximately 70% of PCa patients in the United States are diagnosed at a localized stage. These low-risk PCa patients have a nearly 100% 5- and 10-year survival rate, but the rate drops to only 34% for those with distant metastasis [2,3]. This major difference in survival highlights the different needs of various stages of PCa patients. For low-risk localized PCa patients, constant disease monitoring or active surveillance with regular PSA tests and biopsy exams is often suggested. For clinically active PCa, treatment options include surgery, radiation, and hormonally directed therapy, all of which can often lead to adverse side effects. Given the diverse PCa medical care approaches and their potential side effects, assessing quality of life (QoL) becomes crucial. QoL encompasses various dimensions, including physical, emotional, and social well-being [4], making it a critical measure for PCa patients. QoL reflects PCa patients’ overall well-being and is linked to PCa progression and mortality [5,6,7]. Several factors (such as age, race, treatment modalities, and cancer stage) influencing QoL among PCa patients have been reported [8,9,10,11].

Because of PCa disease itself and its related disease monitoring and treatments, PCa patients often experience fatigue. Fatigue is one of the most common and distressing side effects for PCa patients, affecting 40% of PCa patients in general and up to 90% of those with advanced-stage PCa [12,13]. Fatigue is persistent exhaustion from reduced physical and mental capacity, leading to ongoing tiredness not relieved by rest [14,15]. It can affect daily activities for healthy people and individuals with diseases such as PCa [15]. The link between fatigue and QoL in PCa patients is complicated and multi-dimensional [16].

Despite existing research, several gaps remain. Most QoL studies involving PCa patients are clinic-based and feature small sample sizes. Additionally, many focus on general QoL, often neglecting the effects of fatigue on physical and mental health. The roles of health-related behaviors, such as smoking and alcohol intake, alongside clinical factors tied to PCa, in influencing QoL outcomes are still unclear. To address these gaps, this study aims to evaluate the fatigue associated with QoL and its related health measures (physical and mental health) among PCa patients using the large-scale All of Us cohort.

## 2. Materials and Methods

### 2.1. Study Design

**Figure 1:**
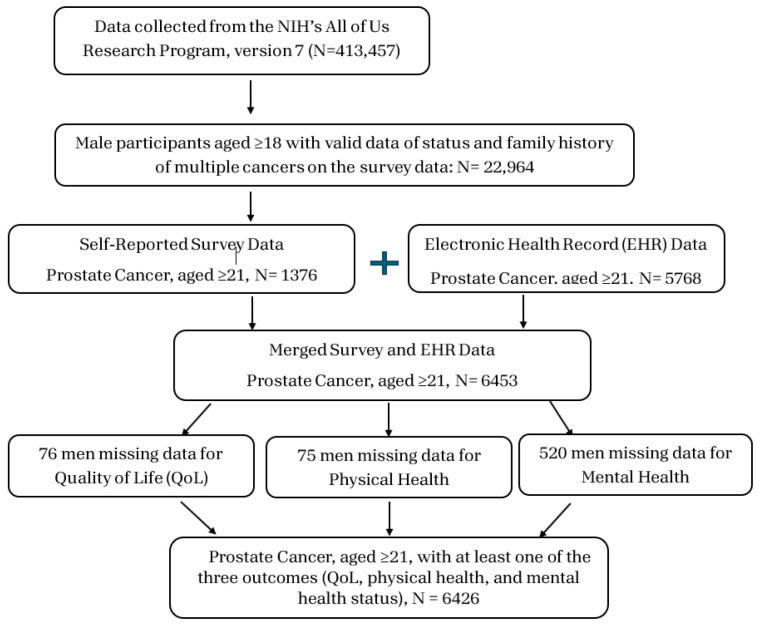
Study population selection procedure. Note: N: sample size.

This is a cross-section study based on secondary data analyses using the All of Us Research Program data conducted by the National Health Institute in the United States (US). This study used 6426 PCa patients based on the All of US Curated Data Repository version 7 (CDRv7) Controlled Tier data, which enrolled participants between May 2018 and 1 July 2022. The inclusion criteria for this study were PCa patients aged 21 and above with at least one of the three outcomes (QoL, physical health, and mental health status). PCa patients were selected based on self-reported status and electronic health record (EHR) data. The flowchart of the study population selection is listed in Figure 1. The All of Us Research Program aims to collect and study data from more than one million people aged 18 or older living in the US to accelerate biomedical research and improve health. The All of Us program started in January 2015 and opened for enrollment in May 2018 [17].

### 2.2. Data Quality

This study thoroughly checked the data management and variable recoding procedures to ensure data quality and accuracy. In addition, the All of Us Research Program had rigorous data quality control approaches to ensure data quality for various data sources. The Observational Medical Outcomes Partnership Common Data Model was applied to standardize EHR data. Then, the All of Us program cleaned the data and used different access tiers (Public, Registered, and Controlled Tier) containing different variables and privacy protection [18].

### 2.3. Measures

The 3 outcomes (QoL, physical health, and mental health status) were measured using the following questions in the All of Us survey. The QoL status was measured using the question, “In general, would you say your quality of life is (poor, fair, good, very good, or excellent)?” Physical health was measured using the question, “In general, how would you rate your physical health?”, and mental health was collected using the question “In general, how would you rate your mental health, including your mood and your ability to think?” The responses to the three questions were based on a 5-item Likert scale: poor, fair, good, very good, and excellent. In this study, the responses to the outcomes were grouped into 2 groups: “poor” for poor or fair and “good” for good, very good, or excellent.

The variables we considered as predictors in this study included 5 demographic factors (age, race, marital status, education, and annual household income), 5 general health-related factors (Body Mass Index (BMI), smoking, alcohol intake in the past year, social function ability, and fatigue level in the past 7 days), and 3 PCa clinical factors (Prostate-Specific Antigen (PSA, ng/mL), current PCa therapy, and age at PCa diagnosis). The details of other health-related measures used for this study are listed below. The BMI categories are as follows: underweight (BMI < 18.5 kg/m^2^); normal weight (18.5–24.9); overweight (25.0–29.9); and obesity (≥30.0). The BMI variable used in this study was derived from the height and weight variables, which are a part of the Physical Measurements module of the All of Us Research Program data. These data had two sources: EHR data and data collected during the in-person visit by trained personnel during participants’ initial visits in the affiliated organizations with the All of Us Research Program [19]. Smoking status was collected using two questions: “Have you smoked at least 100 cigarettes in your entire life? (There are 20 cigarettes in a pack.)?” and “Do you now smoke cigarettes every day, some days, or not at all?”. The never smokers were defined as those who did not smoke 100 cigarettes in their entire life, and the former smokers were those who smoked 100 or more cigarettes in their entire life but did not smoke now. The current smokers were those who smoked 100 cigarettes in their entire life and smoked now. Alcohol intake was measured using two questions: “In your entire life, have you had at least 1 drink of any kind of alcohol, not counting small tastes or sips? (By a “drink,” we mean a can or bottle of beer, a glass of wine or a wine cooler, a shot of liquor, or a mixed drink with liquor in it.)” and “How often did you have a drink containing alcohol in the past year?” for those who answered yes in the first question. In addition, social function ability was assessed by asking, “In general, how well do you carry out your usual social roles? (This includes activities at home, at work, and in your community, as well as responsibilities as a parent, child, spouse, employee, friend, etc.)” Responses were measured on the 5-item Likert scale. We classified social function as “poor” for responses of fair or poor, and as “good” for responses of good/very good/excellent.

Fatigue level was evaluated with the question, “In the past 7 days, how would you rate your fatigue?” using the 5-item Likert scale. Due to a small number of participants with a very severe fatigue level, we converted fatigue from 5 to 3 levels: low (none or mild), moderate, and high (severe or very severe). The PCa therapy status was a self-reported measure using the question, “Are you currently prescribed medications and/or receiving treatment for prostate cancer?”. Prostate-Specific Antigen (PSA in ng/mL) levels measured in the blood were obtained based on participants’ EHR data. PSA is a protein produced by both normal and malignant cells of the prostate gland. For PCa patients, PSA is commonly used to monitor PCa progression. A high PSA level (>10 ng/mL) often indicates the extent and aggressiveness of the disease, and a PSA level above 4.0 ng/mL is considered abnormal [20]. In this study, the age at PCa diagnosis variable was initially recorded in a wide interval (18–64, 65–74, and 75+), so the exact duration of PCa cannot be calculated. Thus, we used age at PCa diagnosis (<65 and ≥65) instead.

All survey data were collected during enrollment. The latest measurements were used for BMI and PSA. For identifying PCa patients using the EHR data, the PCa status was determined based on all available EHR data collected over time. In the All of Us Research Program, EHR data were submitted either by healthcare provider organizations or directly from participants via their online portals [21]. The amount and duration of EHR data differed by participants and medical events.

### 2.4. Statistical Analyses

These variables of interest in the whole study group and the status of the 3 outcomes (QoL, physical health, and mental health) were summarized using descriptive statistics. The variables of interest are listed in Table 1, including demographic, general health-related, and PCa clinical factors. Most PCa patients had health insurance (98.4%); therefore, health insurance was not considered for further analyses. The factors associated with the binary outcome were tested using a *t*-test for continuous variables and the chi-square test for categorical variables. In addition, the associations between each factor and the outcome were tested using univariate and multivariable logistic models. The variable selection of each outcome’s final multivariable logistic model was based on the stepwise selection with the entry and removal alpha level of 0.05. In addition to the primary predictor of fatigue level, other adjusted factors are race, marital status, income, smoking, social function ability, physical health, and mental health for quality of life; age, race, marital status, education, income, BMI, smoking, alcohol intake, and social function ability for physical health; and age, marital status, education, income, and social function ability for mental health. Moreover, we tested 3 PCa clinical-related factors, including PSA, age at PCa diagnosis, and self-reported PCa therapy status (yes/no). However, only a subset of 2933 PCa patients (46%) had these clinical factors, so separated models were performed by considering these PCa clinical factors.

The odds ratios (ORs) and the corresponding 95% confidence intervals (CIs) of these factors were calculated. The effect size of the predictors was based on the OR values in the models. An OR of ≤1.5 indicates a small effect size, 1.6–2.5 represents a medium effect size, 2.6–4 signifies a large effect size, and >4 denotes a very large effect size [22]. The models’ performance was evaluated using the area under the receiver operating characteristic curve (AUC). The AUC value of 0.5–1.0 (no better than chance to perfect discrimination) summarizes the model’s ability to distinguish between the two study groups, such as individuals with good/poor QoL physical or mental health. AUC values ≥0.80 are considered practically or clinically useful, and ≥0.90 are considered excellent discrimination [23]. The statistically significant level of this study was 0.05.

## 3. Results

**Figure 2:**
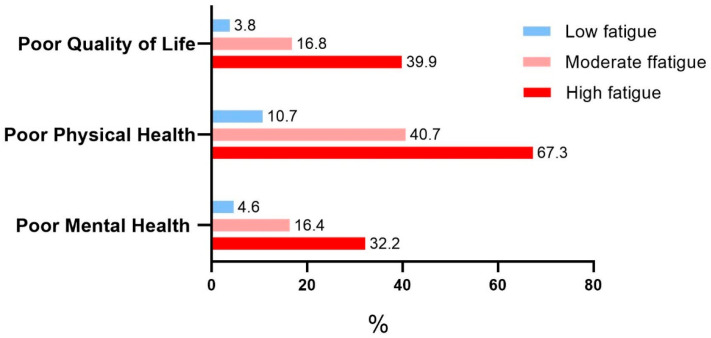
Quality of life, physical health, and mental health status by fatigue level for prostate cancer patients. Note: associations between fatigue levels and all three outcomes were significant (*p* < 0.001).

There were 6426 PCa patients included in this All of Us study. Among them, 558 (8.8%) reported poor QoL, 1324 (20.9%) reported poor physical health, and 534 (9.0%) reported poor mental health. There were 4488 (70.5%), 1563 (24.5%), and 315 (5.0%) PCa patients with low, moderate, and high fatigue levels, respectively (Supplementary Table S1). The characteristics of PCa patients by QoL status are shown in Table 1 and Figure 2. Based on bivariate analyses, all testing demographic and general health-related factors were significantly (*p* < 0.001) associated with QoL. While poor QoL prevalence was 8.8% for PCa patients overall, some sub-groups had a much higher risk of poor QoL. As shown in Figure 2, 39.9% of PCa patients with a high fatigue level and 16.8% with a moderate fatigue level reported poor QoL, but only 3.8% with a low fatigue level had poor QoL (*p* < 0.001). Those with poor social function ability tended to have poor QoL (50.9%). In addition, the results showed that younger individuals; African Americans; those who were never married, divorced, widowed, or separated; and those with lower education levels and low income reported poor QoL. All differences were statistically significant (*p* < 0.001). PCa patients with poor QoL were younger on average than those with high QoL (72.4 vs. 75.9 years). As for race, African American men tended to have poor QoL (18.0%) compared with White (5.9%) and other race (13.9%) groups. Those who were never married, divorced, widowed, or separated had a higher chance (16.8%) of poor QoL than those who married or lived with a partner (5.5%). For education, PCa patients with less than a high school or high school degree tend to report poor QoL (19.8%) compared with those with college or advanced degrees (8.3% and 4.2%, respectively). In addition, PCa patients with low annual household income (<USD 50,000) had poorer QoL (18%) than those with a higher household income (3.7% for ≥USD 100,000). Other significant factors included BMI, smoking status, and alcohol consumption (all *p*-values < 0.001).

PCa patients’ characteristics by physical health and mental health status are shown in Table 2 and Figure 2. Fatigue level and social function ability play an important role in physical health. Those with a high fatigue level (67.3%) and poor social function ability (72.9%) had a high risk of poor physical health (*p* < 0.001). In addition, patients with poor physical health were generally younger (mean age 73.7 years vs. 76.1 for poor and good physical health, respectively), African American (31.8% with poor physical health), and other races (30.5%); never married, divorced, widowed, or separated (30.3%); and had low education (37.4% for high school or less) and low annual household income (32.9%). In addition, health-related factors (BMI, smoking status, and alcohol intake) were all significantly associated with poor physical health (all *p* < 0.001). PCa patients who were obese (26.9%), current smokers (39.4%), and infrequent alcohol drinkers (drink ≤ 1 time per month, 27.2%) had a high chance of poor physical health. The three PCa clinical factors were also associated with poor physical health. PCa patients with a high PSA level (≥10 ng/mL, 26.9%), with PCa therapy (22.4%), and diagnosed PCa at an age less than 65 years old (18.7%) tended to have poor physical health.

For mental health, risk factors associated with poor mental health were similar to physical health (Table 2 and Figure 2). PCa patients with poor mental health were generally younger (mean age 72.3 years vs. 75.8 for poor and good mental health, respectively), African American (15.9% with poor mental health), and other races (12.2%); never married, divorced, widowed, or separated (15.0%); and had low education (18.7% for high school or less) and low annual household income (15.2%). In addition, PCa patients who were current smokers (21.3%) and infrequent alcohol drinkers (11.5%) had a high chance of having poor mental health. For BMI, PCa patients who were underweight/normal (10.9%) and obese (10.1%) were likely to report poor mental health. Those with poor social function ability (44.4%) and with a high fatigue level (32.2%) had a high risk of poor mental health (*p* < 0.001). Clinical factors like PSA levels and current PCa therapy status did not significantly affect mental health (*p* = 0.903 and 0.131, respectively). Moreover, PCa patients diagnosed with PCa at a younger age tended to have poor mental health (8.2%). The prevalence of poor QoL, physical health, and mental health by fatigue status is summarized in Figure 2.

The factors associated with poor QoL for PCa patients, with and without adjusting for other factors, are summarized in Table 3. Fatigue was significantly associated with QoL in both univariate and multivariable models. PCa patients with a moderate or high fatigue level in the past 7 days reported a significantly poorer QoL than those with a low fatigue level (OR = 1.46, *p* = 0.007 for moderate fatigue level; OR = 2.33, *p* < 0.001 for high fatigue level in the multivariable model). Based on the multivariable model, marital status and income were also strong predictors, with never married, divorced, widowed, or separated (OR = 1.65, *p*< 0.001) and low household income patients (OR = 2.12, *p* < 0.001) more likely to report poorer QoL. Patients who were former smokers had a lower risk of poor QoL (OR = 0.66, *p* < 0.001). Furthermore, PCa patients with poor social function ability (OR = 3.07, *p* < 0.001) tended to have poor QoL. Both physical health and mental health were also strong predictors for QoL. PCa patients with poor physical health (OR = 14.69, *p* < 0.001) and poor mental health (OR = 4.79, *p* < 0.001) tended to have poor QoL. Furthermore, none of the PCa clinical factors (PSA, PCa diagnosis age, and PCa therapy) were associated with QoL after considering other factors listed in Table 3 based on the sub-group analyses.

The factors associated with poor physical health, with and without adjusting for other factors, are summarized in Table 4. Fatigue level significantly impacted physical health in both univariate and multivariable models. PCa patients with a moderate or high fatigue level were likely to report poor physical health (OR = 4.19, *p* < 0.001 for moderate level and OR = 8.20, *p* < 0.001 for high level) after adjusting for demographics, health behavior, and other factors listed in Table 4. In addition, PCa patients with a poor ability to carry out regular social roles had poor physical health (OR = 6.20, *p* < 0.001) based on the adjusted model. Moreover, PCa patients who were never married, divorced, widowed, or separated; low educated; low income; formerly or currently smoking; and had less alcohol intake tended to have poor physical health. For sub-group analyses with 2933 PCa patients with PCa clinical data (Supplementary Table S2), patients who currently had PCa therapy (OR = 1.30, *p* = 0.030) had a higher risk of poor physical health than those without therapy after adjusting for other factors.

The factors associated with poor mental health, with and without adjusting for other factors, are summarized in Table 5. PCa patients with a moderate or high fatigue level were more likely to report poor mental health than others with a low fatigue level (OR = 2.64, *p* < 0.001 for moderate level and OR = 3.62, *p* < 0.001 for high level). PCa patients with poor social function ability tended to report poor mental health (OR = 6.63, *p* < 0.001). In addition, PCa patients who were younger (OR = 0.97 per 1 year, *p* < 0.001); never married, divorced, widowed, or separated (OR = 1.41, *p* = 0.003); and had low education (OR = 0.53, *p* < 0.001 and 0.62, *p* = 0.003 for college and advanced degree compared with high school or less) were likely to report poor mental health. Furthermore, we were interested in evaluating whether PCa clinical factors and age were associated with fatigue. As shown in Supplementary Table S3, age was significantly associated with fatigue (*p* < 0.001), and those with a high fatigue level were younger (72.3 for the high level vs. 75.7 for both low and moderate levels). PSA level was not significantly associated with fatigue level (*p* = 0.094). PCa patients with PCa-related therapy currently tended to have a higher fatigue level than those without therapy (*p* < 0.001). In addition, PCa patients who were diagnosed with PCa at a younger age (<65 years old) were likely to report a higher fatigue level than those who were diagnosed with PCa at ≥65 years old (*p* = 0.002).

In summary, the impact of fatigue level on QoL, physical health, and mental health, adjusting for other factors, was highly significant (*p* < 0.001) and had a medium-to-very-large effect size. Regarding the magnitude of fatigue impact, fatigue greatly impacts physical health (OR = 8.20). The impact of fatigue on mental health is considered large (OR = 3.62) and on QoL is considered medium (OR = 2.33). It is worth mentioning that all multivariable models had an AUC greater than 0.8, which is considered clinically useful. The AUC values were 0.945, 0.820, and 0.814 for QoL, physical health, and mental health, respectively. The AUC for physical health with PCa treatment was 0.818.

## 4. Discussion

Our findings revealed that high fatigue levels were significantly associated with poor QoL, physical health, and mental health in PCa patients. All associations were highly significant (*p* < 0.001) and with medium-to-large effect sizes. Fatigue had the greatest impact on physical health (OR = 8.20), followed by mental health (OR = 3.62) and QoL (OR = 2.33) after adjusting for other factors. In addition to fatigue, social function ability also plays a critical role in terms of the three QoL-related outcomes. PCa patients with poor social function ability also had poor QoL, physical health, and mental health. Social function ability had the greatest impact on mental health (OR = 6.63), followed by physical health (OR = 6.20) and QoL (OR = 3.07) after adjusting for other factors. Among the PCa clinical factors, only PCa therapy was significantly associated with poor physical health but not with QoL and mental health. While PSA levels are commonly used to monitor PCa progression, PSA was not significantly associated with the three outcomes.

Regarding the prevalence of QoL-related outcomes, our results showed that 8.8% of PCa patients reported poor QoL, 20.9% reported poor physical health, and 9.0% reported poor mental health. Two studies used the QLQ-C30 questionnaire to measure QoL for PCa patients and reported that 31–32% of PCa patients reported poor global QOL, 18–37% reported poor physical function, and 34–55% reported poor emotional function [24,25]. Compared to these two PCa studies, the prevalence of the QoL-related outcomes in this study is lower. This could be due to different measuring methods and different features of PCa patients, such as tumor stage and treatment types [24]. Studies indicate that the side effects of PCa treatments, such as fatigue, urinary incontinence, and sexual dysfunction, can impact QoL for many PCa patients [26].

Although QoL measures are subjective, studies have shown that high QoL measured at various times and improvements in QoL are significantly associated with objective clinical outcomes, including overall survival and progress-free survival, for PCa patients. A meta-analysis study showed QoL can predict overall survival for many cancer types, including PCa [7]. In the same study, this QoL was measured at various time points (pre-treatment, post-treatment, and palliative care), significantly impacting overall survival. These associations between QoL and overall survival varied by PCa treatment types. Notably, patients with poor QoL tend to experience worse survival rates, particularly those with active surveillance and high-dose radiotherapy, although this association is not significant in patients with a radical prostatectomy [24]. Further supporting these findings, another study based on two large-scale phase III clinical trials with metastatic castration-resistant PCa, an advanced form of PCa, measured QoL multiple times before, during, and after treatments [5]. The results showed that both high baseline QoL and improvements in QoL based on longitudinal data significantly increased overall survival and radiographic progression-free survival for these PCa patients [5].

Additionally, our findings revealed that fatigue contributes to QoL beyond the impacts of physical and mental health, emphasizing its role as an independent factor. Fatigue affects PCa patients’ health in multiple dimensions, such as increasing sleep disturbance, impairing cognitive function, reducing physical ability, inability to work, and decreasing social function activities [12,27,28,29]. In this study, approximately 30% of PCa patients had a moderate or high fatigue level, aligning with findings from other studies. Cancer-related fatigue is a big burden for cancer and its treatments [13]. A meta-study estimated the cancer-related fatigue prevalence for PCa patients as 40% with a range of 17–82%, affected by various factors, such as treatment types [13]. For example, it has been shown that patients with androgen deprivation therapy (ADT) and radiotherapy had a higher chance of fatigue than those with radical prostatectomy [30]. For reducing fatigue in PCa patients, several non-pharmacological interventions, such as physical activity (aerobic and resistance exercise), education, and cognitive behavioral therapy, have shown promising results [12,31].

While fatigue prominently affects QoL, it is also essential to acknowledge that social function significantly affects QoL and physical and mental health. These results were also supported by other studies. Higher levels of social support are often related to better social functioning abilities, indicating that strong social networks can help patients maintain their social roles, reduce psychological distress, and improve their overall QoL for PCa patients [32,33]. Some interventions, such as patient support groups [34] and religious or spiritual coping strategies [35], have been shown to bolster social support, enhance mental health, and ultimately improve QoL for PCa patients. Moreover, demographic factors also played a significant role in QoL outcomes in our study. We identified PCa patients who are African American, lower educated and lower income, have specific marital status (never married, divorced, widowed, or separated), and are current smokers tend to have poor QoL. These findings align with previous research that PCa patients who are African American, have lower education levels, impaired mental health, comorbidities, higher clinical stages, higher Gleason scores, greater cancer severity, and undergo neoadjuvant hormonal therapy tended to have poor health-related QoL [36].

For factors associated with QoL, we were also interested in knowing whether PCa progression-related factors also impacted QoL. This study showed that PSA levels and age at PCa diagnosis did not affect QoL and mental health, although they have been shown to be linked to PCa progression. Only current PCa therapy was significantly associated with physical health. PSA levels are crucial for detecting and monitoring the progression of PCa. Elevated PSA levels are strongly associated with an increased risk of disease progression and poorer clinical outcomes. PSA testing is recognized as a primary tool for monitoring PCa progression, especially in active surveillance for low- to intermediate-risk patients, according to several guidelines, including those from the American Urological Association (DUA) and the National Comprehensive Cancer Network (NCCN). These guidelines recommend performing PSA tests every 3 to 6 months [37]. For age at PCa diagnosis, it has been shown that PCa patients diagnosed at an older age tended to have poor disease prognosis [38,39]. Additionally, medications and treatments for PCa have been reported to impact QoL in several studies [24,40]. PCa patients who are on active surveillance or have undergone radical prostatectomy often report a better QoL compared to those receiving high-dose radiotherapy [24].

It is worth mentioning that the multivariable logistic models for QoL, physical health, and mental health had an excellent performance, with AUC values ranging from 0.8 to 0.9, which are considered clinically useful [23]. These findings can help clinicians identify PCa patients at a higher risk of poor QoL, physical health, or mental health so clinicians can proactively address their needs, ultimately improving patient care and outcomes. For example, pre-treatment evaluations of QoL can help patients and physicians choose the most appropriate interventions, such as active surveillance, radiation therapy, or surgical options. Additionally, QoL assessments conducted after treatment and during palliative care can guide decisions regarding necessary rehabilitation interventions [7,24]. Thus, multiple QoL measures should be conducted throughout cancer care for PCa patients to monitor QoL changes over time [41,42].

This study has several strengths. First, this study has a large sample size of PCa patients, which enhances the reliability of the findings. Second, both self-reported measures and objective EHR data were used. We utilized self-reported and EHR data to identify PCa patients, which allowed us to include a broader range of participants. Furthermore, we incorporated PSA levels obtained from EHR data to enhance data accuracy. Third, this study considered factors from various domains, such as QoL, physical health, mental health, health behaviors, psychological factors, and PCa clinical factors. However, there are limitations to acknowledge. First, the three outcomes, fatigue, and social function used in this study were based on one question that may not measure these concepts comprehensively. Second, physical activity levels, one of the important factors for QoL, were not included due to data availability. Third, the clinical features (such as tumor stage) and treatment information (treatment type and history) for the PCa participants were unknown, so their impact could not be evaluated. Fourth, due to the cross-sectional nature of this study, causal inferences between fatigue and outcome variables (QoL and physical and mental health) cannot be made. The observed associations of fatigue and these outcomes may reflect bidirectional or reverse relationships. Fifth, fatigue may share symptomatic overlap with poor physical and mental health, which may partially explain the observed associations. However, evaluating the associations between fatigue and QoL-related outcomes is essential for a comprehensive understanding of fatigue’s impact on overall health, leading to improved diagnosis and intervention strategies. Sixth, some potential side effects of PCa treatments (such as urinary incontinence and sexual dysfunction), which may affect the QoL-related outcomes, were not taken into consideration in this study. Seventh, this study used the All of Us Research Program data, which enrolled participants from 2018 to 2022, so it was seriously affected by the COVID-19 pandemic. Because of this pandemic, all program-related in-person activities, including enrollment, were paused from March 2020 to February 2022, reducing the number of enrollments and data collection [43]. Lastly, it is important to note that recall biases may affect the accuracy of the self-reported information.

## 5. Conclusions

This study showed that a high fatigue level was significantly associated with poor QoL, physical health, and mental health for PCa patients. This finding highlights the importance of addressing fatigue in PCa patients’ supportive care. Because of the long survival time for most PCa, the focus on improving QoL is critical. Several interventions (such as physical activity and education) have been shown to effectively reduce fatigue levels for PCa patients. Thus, fatigue management should be considered to enhance QoL and overall well-being and health outcomes for PCa patients.

## Figures and Tables

**Table 1:** Prostate cancer patients’ characteristics overall and by quality of life status.

	All	Quality of Life		
	N (%) Mean ± SD ^1^	Good N (%) Mean ± SD ^1^	Poor N (%) Mean ± SD ^1^	*p*-Value ^2^
Total	6426	5792 (91.2)	558 (8.8)	-
**Demographic factors**				
Age	75.9 ± 8.4	75.9± 8.3	72.4 ± 9.3	<0.001
Race				
White	4751 (81.5)	4428 (94.1)	276 (5.9)	<0.001
African Americans and others	1077 (18.5)	875 (82.8)	182 (17.2)	
Missing	598	489 (83.0)	100 (17.0)	
Marital status				
Married/living with partner	4553 (71.9)	4260 (94.5)	246 (5.5)	<0.001
Never married/divorced/widowed/separated	1783 (28.1)	1465 (83.2)	295 (16.8)	
Education				
≤High school graduate	1032 (16.4)	808 (80.2)	200 (19.8)	<0.001
Some college/college graduate	2928 (46.4)	2659 (91.7)	241 (8.3)	
Advanced degree	2345 (37.2)	2230 (95.8)	97 (4.2)	
Annual household income				
<50,000	1625 (30.2)	1311 (82.0)	287 (18.0)	<0.001
≥50,000	3748 (69.8)	3578 (96.3)	138 (3.7)	
Missing	1053	903 (87.2)	133 (12.8)	
**General health-related factors**				
BMI				
Underweight/normal	1489 (24.4)	1326 (90.1)	146 (9.9)	<0.001
Overweight	2643 (43.4)	2432 (93.0)	182 (7.0)	
Obesity	1961 (32.2)	1728 (89.4)	204 (10.6)	
Missing	333	306 (92.2)	26 (7.8)	
Smoking status				
Never	3229 (51.4)	2952 (92.4)	243 (7.6)	<0.001
Former	2566 (40.9)	2355 (92.7)	185 (7.3)	
Current	484 (7.7)	362 (76.2)	113 (23.8)	
Drink containing alcohol in the past year				
Never/monthly or less	2865 (45.2)	2497 (88.3)	331 (11.7)	<0.001
2–4 times/month or 2–3 times/week	1074 (17.0)	972 (91.6)	89 (8.4)	
≥4 times/week	2396 (37.8)	2254 (94.9)	122 (5.1)	
Social function ability				
Poor	529 (8.3)	257 (49.1)	267 (50.9)	<0.001
Good	5829 (91.7)	5477 (95.0)	288 (5.0)	
**PCa clinical factors**				
Prostate-Specific Antigen (PSA, ng/mL)				
<4	3014 (73.6)	2723 (91.4)	256 (8.6)	0.203
4–10	745 (18.2)	675 (92.2)	57 (7.8)	
≥10	336 (8.2)	297 (88.9)	37 (11.1)	
Missing	2231	2097 (91.0)	208 (9.0)	
Current PCa therapy				
No	1999 (68.2)	1890 (95.3)	93 (4.7)	0.014
Yes	934 (31.8)	864 (93.1)	64 (6.9)	
Age at PCa diagnosis				
<65	1673 (57.1)	1553 (93.5)	108 (6.5)	0.002
≥65	1259 (42.9)	1200 (96.1)	49 (3.9)	

^1^ Standard deviation; N: sample size. ^2^ *t*-test for age and chi-square test for categorical variables, which did not include the missing group.

**Table 2:** Prostate cancer patients’ characteristics by physical health and mental health status.

	Physical Health			Mental Health		
	Good N (%) Mean ± SD ^1^	Poor N (%) Mean ± SD ^1^	*p*-Value ^2^	Good N (%) Mean ± SD ^1^	Poor N (%) Mean ± SD ^1^	*p*-Value ^2^
Total	5027 (79.2)	1324 (20.9)	-	5372 (91.0)	534 (9.0)	-
**Demographic factors**						
Age	76.1 ± 8.3	73.7± 8.8	<0.001	75.8 ± 8.4	72.3 ± 8.9	<0.001
Race						
White	3900 (82.8)	810 (17.2)	<0.001	4042 (92.9)	308 (7.1)	<0.001
African Americans and others	719 (68.2)	335 (31.8)		831 (84.1)	157 (15.9)	
Missing	408 (69.5)	179 (30.5)		499 (87.9)	69 (12.2)	
Marital status						
Married/living with partner	3739 (83.0)	766 (17.0)	<0.001	3890 (93.4)	274 (6.6)	<0.001
Never married/divorced/widowed/separated	1226 (69.7)	533 (30.3)		1409 (85.0)	249 (15.0)	
Education						
≤High school graduate	635 (62.6)	379 (37.4)	<0.001	789 (81.3)	181 (18.7)	<0.001
Some college/college graduate	2303 (79.6)	591 (20.4)		2508 (92.1)	216 (7.9)	
Advanced degree	2010 (86.2)	321 (13.8)		1976 (94.1)	124 (5.9)	
Annual household income						
<50,000	1073 (67.2)	525 (32.9)	<0.001	1288 (84.8)	231 (15.2)	<0.001
≥50,000	3181 (85.6)	535 (14.4)		3206 (94.2)	196 (5.8)	
Missing	773 (74.5)	264 (25.5)		878 (89.1)	107 (10.9)	
**General health-related factors**						
BMI						
Underweight/normal	1159 (78.8)	311 (21.2)	<0.001	1198 (89.1)	146 (10.9)	0.001
Overweight	2184 (83.7)	425 (16.3)		2241 (92.5)	183 (7.5)	
Obesity	1417 (73.1)	522 (26.9)		1626 (89.9)	182 (10.1)	
Missing	267 (80.2)	66 (19.8)		307 (93.0)	23 (7.0)	
Smoking status						
Never	2634 (82.4)	561 (17.6)	<0.001	2714 (92.6)	218 (7.4)	<0.001
Former	1992 (78.3)	552 (21.7)		2185 (91.6)	200 (8.4)	
Current	287 (60.6)	187 (39.4)		359 (78.7)	97 (21.3)	
Drink containing alcohol in the past year						
Never/monthly or less	2060 (72.8)	770 (27.2)	<0.001	2348 (88.5)	305 (11.5)	<0.001
2–4 times/month or 2–3 times/week	843 (79.5)	217 (20.5)		909 (91.8)	81 (8.2)	
≥4 times/week	2058 (86.7)	316 (13.3)		2045 (93.8)	136 (6.2)	
Social function ability						
Poor	142 (27.1)	382 (72.9)	<0.001	286 (55.6)	228 (44.4)	<0.001
Good	4835 (83.9)	931 (16.1)		5033 (94.4)	299 (5.6)	
**PCa clinical factors**						
Prostate-Specific Antigen (PSA, ng/mL)						
<4	2371 (79.6)	609 (20.4)	0.003	2493 (90.7)	255 (9.3)	0.903
4–10	606 (82.3)	130 (17.7)		605 (90.6)	63 (9.4)	
≥10	242 (73.1)	89 (26.9)		278 (91.5)	26 (8.5)	
Missing	1808 (78.5)	496 (21.5)		1996 (91.3)	190 (8.7)	
Current PCa therapy						
No	1708 (85.9)	281 (14.1)	<0.001	1741 (93.9)	113 (6.1)	0.131
Yes	719 (77.6)	208 (22.4)		811 (92.4)	67 (7.6)	
Age at PCa diagnosis						
<65	1353 (81.3)	311 (18.7)	0.001	1443 (91.8)	129 (8.2)	<0.001
≥65	1075 (85.9)	177 (14.1)		1109 (95.7)	50 (4.3)	

^1^ Standard deviation; N: sample size. ^2^ *t*-test for age and chi-square test for categorical variables, which did not include the missing group.

**Table 3:** Factors associated with poor quality of life for prostate cancer patients.

	Univariate Model		Multivariable Models ^2^ (n = 5993)	
	OR (95% CI) ^1^	*p*-Value	OR (95% CI) ^1^	*p*-Value
Race				
White	1		1	
African Americans and others	3.34 (2.73, 4.08)	<0.001	1.31 (0.96, 1.77)	0.084
Missing	3.28 (2.56, 4.2)	<0.001	1.98 (1.39, 2.83)	<0.001
Marital status				
Married/living with partner	1		1	
Never married/divorced/widowed/separated	3.49 (2.92, 4.17)	<0.001	1.65 (1.26, 2.15)	<0.001
Annual household income				
<50,000	5.68 (4.59, 7.02)	<0.001	2.12 (1.56, 2.88)	<0.001
≥50,000	1		1	
Missing	3.82 (2.98, 4.9)	<0.001	1.87 (1.32, 2.65)	<0.001
Smoking status				
Never	1		1	
Former	0.95 (0.78, 1.16)	0.645	0.66 (0.50, 0.86)	0.002
Current	3.79 (2.96, 4.86)	<0.001	1.11 (0.76, 1.61)	0.587
Social function ability				
Poor	19.76(16.04, 24.33)	<0.001	3.07 (2.32, 4.07)	<0.001
Good	1		1	
Fatigue level in the past 7 days				
Low	1		1	
Moderate	5.14 (4.19, 6.31)	<0.001	1.46 (1.11, 1.92)	0.007
High	16.91(12.86, 22.24)	<0.001	2.33 (1.59, 3.41)	<0.001
Physical health				
Good	1		1	
Poor	37 (28.69, 47.72)	<0.001	14.69 (10.95, 19.71)	<0.001
Mental health				
Good	1		1	
Poor	17.67(14.33, 21.77)	<0.001	4.79 (3.63, 6.32)	<0.001
Missing	0.47 (0.27, 0.82)	0.008	0.68 (0.34, 1.37)	0.276

^1^ Odds ratio (95% confidence interval). ^2^ The area under the receiver operating characteristic curve (AUC) = 0.945.

**Table 4:** Factors associated with poor physical health for prostate cancer patients.

	Univariate Model		Multivariable Models ^2^ (n = 5905)	
	OR (95% CI) ^1^	*p*-Value	OR (95% CI) ^1^	*p*-Value
Age	0.97 (0.96, 0.97)	<0.001	0.98 (0.97, 0.99)	<0.001
Race				
White	1		1	
African Americans and others	2.24 (1.93, 2.61)	<0.001	1.23 (1.00, 1.52)	0.053
Missing	2.11 (1.75, 2.56)	<0.001	1.31 (1.01, 1.69)	0.041
Marital status				
Married/living with partner	1		1	
Never married/divorced/widowed/separated	2.12 (1.87, 2.4)	<0.001	1.20 (1.01, 1.43)	0.036
Education				
≤High school graduate	1		1	
Some college/college graduate	0.43 (0.37, 0.50)	<0.001	0.66 (0.54, 0.81)	<0.001
Advanced degree	0.27 (0.23, 0.32)	<0.001	0.63 (0.50, 0.80)	<0.001
Annual household income				
<50,000	2.91 (2.53, 3.34)	<0.001	1.25 (1.03, 1.52)	0.025
≥50,000	1		1	
Missing	2.03 (1.72, 2.40)	<0.001	0.95 (0.75, 1.19)	0.634
BMI				
Underweight/normal	1		1	
Overweight	0.73 (0.62, 0.85)	<0.001	0.81 (0.66, 0.98)	0.033
Obesity	1.37 (1.17, 1.61)	<0.001	1.18 (0.97, 1.45)	0.105
Missing	0.92 (0.68, 1.24)	0.588	1.04 (0.73, 1.50)	0.818
Smoking status				
Never	1		1	
Former	1.30 (1.14, 1.48)	<0.001	1.36 (1.16, 1.60)	<0.001
Current	3.06 (2.49, 3.76)	<0.001	1.35 (1.02, 1.79)	0.035
Drink containing alcohol in the past year				
Never/monthly or Less	1		1	
2–4 times/month or 2–3 times/week	0.69 (0.58, 0.82)	<0.001	0.86 (0.70, 1.05)	0.138
≥4 times/week	0.41 (0.36, 0.48)	<0.001	0.59 (0.49, 0.70)	<0.001
Social function ability				
Poor	13.97 (11.38, 17.15)	<0.001	6.20 (4.87, 7.90)	<0.001
Good	1			
Fatigue level in the past 7 days				
Low	1		1	
Moderate	5.74 (4.99, 6.59)	<0.001	4.19 (3.58, 4.89)	<0.001
High	17.20 (13.31, 22.22)	<0.001	8.20 (6.09, 11.04)	<0.001

^1^ Odds ratio (95% confidence interval). ^2^ The area under the receiver operating characteristic curve (AUC) = 0.820.

**Table 5:** Factors associated with poor mental health for prostate cancer patients.

	Univariate Model		Multivariable Model ^2^ (n = 5636)	
	OR (95% CI) ^1^	*p*-Value	OR (95% CI) ^1^	*p*-Value
Age	0.95 (0.94, 0.96)	<0.001	0.97 (0.95, 0.98)	<0.001
Marital status				
Married/living with partner	1		1	
Never married/divorced/widowed/separated	2.51 (2.09, 3.01)	<0.001	1.41 (1.12, 1.77)	0.003
Education				
≤High school graduate	1		1	
Some college/college graduate	0.38 (0.30, 0.46)	<0.001	0.53 (0.41, 0.69)	<0.001
Advanced degree	0.27 (0.21, 0.35)	<0.001	0.62 (0.45, 0.85)	0.003
Annual household income				
<50,000	2.93 (2.40, 3.59)	<0.001	1.30 (1.00, 1.70)	0.055
≥50,000	1		1	
Missing	1.99 (1.56, 2.55)	<0.001	0.93 (0.68, 1.27)	0.649
Social function ability				
Poor	13.42 (10.88, 16.55)	<0.001	6.63 (5.20, 8.45)	<0.001
Good	1		1	
Fatigue level in the past 7 days				
Low	1		1	
Moderate	4.08 (3.34, 4.99)	<0.001	2.64 (2.10, 3.30)	<0.001
High	9.92 (7.48, 13.15)	<0.001	3.62 (2.58, 5.08)	<0.001

^1^ Odds ratio (95% confidence interval). ^2^ The area under the receiver operating characteristic curve (AUC) = 0.814.

## Data Availability

This study used data from the All of Us Research Program’s Controlled Tier Dataset version 7, available to authorized users on the All of Us Researcher Workbench: https://www.researchallofus.org/data-tools/workbench/ (accessed on 27 April 2025).

## Supplementary Materials

The following supporting information can be downloaded at https://www.mdpi.com/article/10.3390/cancers17091531/s1: Supplementary Table S1: Fatigue levels associated with quality of life, physical health, and mental health; Supplementary Table S2: Factors associated with poor physical health for a sub-group of prostate cancer (PCa) patients (n = 2933) with valid information on PCa clinical factors; Supplementary Table S3: Age and prostate cancer (PCa) clinical factors associated with fatigue levels.
