# Supplementary material for: Fatigue Level Associated with Quality of Life for Prostate Cancer Patients: Results from the All of Us Research Program
Source: Cancers (Basel). 2025 Apr 30;17(9):1531. doi: 10.3390/cancers17091531 (PMC12071964; doi:10.3390/cancers17091531)
Supplement: Supplementary file 1 [file cancers-17-01531-s001.zip › cancers-3591184-supplementary.pdf]

Supplementary Table S1. Fatigue levels associated with quality of life, physical health, and mental health for prostate cancer patients

	All[1]	Quality of life[1]		Physical health[1]		Mental health[1]	
	N (%)	Good<br>N (%)	Poor<br>N (%)	Good<br>N (%)	Poor<br>N (%)	Good<br>N (%)	Poor<br>N (%)
Fatigue level in the past 7 days							
Low	4488 (70.5)	4273 (96.2)	168 (3.8)	3967 (89.3)	475 (10.7)	3878 (95.4)	186 (4.6)
Moderate	1563 (24.5)	1281 (83.2)	259 (16.8)	916 (59.3)	629 (40.7)	1242 (83.6)	243 (16.4)
High	315 (5.0)	188 (60.1)	125 (39.9)	101 (32.7)	208 (67.3)	206 (67.8)	98 (32.2)

[1] All p-values<0.001, N: sample size

Supplementary Table S2. Factors associated with poor physical health for a sub-group of prostate cancer (PCa) patients (n=2933) with valid information on PCa clinical factors

	Univariate model		Multivariable models[2] (n=2827)	
	OR (95% CI)[1]	p-value	OR (95% CI)[1]	p-value
Age	0.96 (0.95, 0.98)	<0.001	0.97 (0.96, 0.98)	<0.001
BMI				
Underweight/ Normal	1		1	
Overweight	0.87 (0.65, 1.16)	0.340	0.77 (0.55, 1.08)	0.134
Obesity	2.11 (1.6, 2.79)	<0.001	1.37 (0.99, 1.91)	0.059
missing	1.5 (1.03, 2.17)	0.033	1.19 (0.77, 1.86)	0.434
Smoking status				
Never	1		1	
Former	1.44 (1.18, 1.77)	<0.001	1.59 (1.24, 2.02)	<0.001
Current	3.15 (2.09, 4.75)	<0.001	1.72 (1.03, 2.86)	0.037
Drink containing alcohol in the past year				
Never/ monthly or Less	1		1	
2-4 times/ month or 2-3 times/ week	0.59 (0.44, 0.78)	<0.001	0.67 (0.48, 0.94)	0.019
≥4 times/ week	0.41 (0.33, 0.51)	<0.001	0.53 (0.41, 0.69)	<0.001
Social function ability				
Poor	15.43 (10.96, 21.72)	<0.001	6.29 (4.25, 9.3)	<0.001
Good	1		1	
Fatigue level in the past 7 days				
Low	1		1	
Moderate	7.07 (5.65, 8.83)	<0.001	4.89 (3.83, 6.25)	<0.001
High	20.56 (13.51, 31.28)	<0.001	11.75 (7.35, 18.76)	<0.001
Current PCa therapy				
No	1		1	
Yes	1.76 (1.44, 2.15)	<0.001	1.30 (1.03, 1.65)	0.030

[1]Odds ratio (95% confidence interval)

[2]The area under the receiver operating characteristic curve (AUC)=0.821

Supplementary Table S3. Age and prostate cancer (PCa) clinical factors associated with fatigue levels

**PCa clinical factors**	Fatigue level in the past 7 days			
	Low<br>N (%)<br>mean± SD[1]	Moderate<br>N (%)<br>mean± SD[1]	High<br>N (%)<br>mean± SD[1]	p-value[2]
Age	75.7± 7.5	75.7± 8.3	72.3± 8.4	<0.001
Prostate-Specific Antigen (PSA, ng/mL)				
<4	937 (73.8)	293 (23.1)	40 (3.2)	0.094
4-10	216 (79.4)	49 (<20.0)	<20 (<5)	
≥10	75 (69.4)	26 (<25.0)	<20 (<10)	
Missing	970 (75.6)	257 (20.0)	56 (4.4)	
Current PCa therapy				
No	1561 (78.4)	377 (18.9)	54 (2.7)	<0.001
Yes	629 (67.5)	247 (26.5)	56 (6.0)	
Age at PCa diagnosis				
< 65	1214 (72.8)	376 (22.6)	77 (4.6)	0.002
≥ 65	974 (77.6)	248 (19.8)	33 (2.6)	

[1]Standard deviation; , N: sample size

[2]t-test for age and Chi-square test for categorical variables, which did not include the missing group
